# Guidelines for Perioperative Management of the Diabetic Patient

**DOI:** 10.1155/2015/284063

**Published:** 2015-05-19

**Authors:** Sivakumar Sudhakaran, Salim R. Surani

**Affiliations:** ^1^Texas A&M Health Science Center, 8447 State Highway 47, Bryan, TX 77807, USA; ^2^Division of Pulmonary, Critical Care & Sleep Medicine, Texas A&M Health Science Center, Corpus Christi, 1177 West Wheeler Avenue, Suite 1, Aransas Pass, TX 78336, USA

## Abstract

Management of glycemic levels in the perioperative setting is critical, especially in diabetic patients. The effects of surgical stress and anesthesia have unique effects on blood glucose levels, which should be taken into consideration to maintain optimum glycemic control. Each stage of surgery presents unique challenges in keeping glucose levels within target range. Additionally, there are special operative conditions that require distinctive glucose management protocols. Interestingly, the literature still does not report a consensus perioperative glucose management strategy for diabetic patients. We hope to outline the most important factors required in formulating a perioperative diabetic regimen, while still allowing for specific adjustments using prudent clinical judgment. Overall, through careful glycemic management in perioperative patients, we may reduce morbidity and mortality and improve surgical outcomes.

## 1. Introduction

Diabetes has classically been defined as a group of metabolic diseases characterized by hyperglycemia due to defects in insulin secretion, insulin action, or a combination of both [1]. The vast majority of diabetic cases can be classified as either type 1 or type 2 diabetes. Type 1 diabetes is generally due to *β*-cell destruction leading to absolute insulin deficiency. This form accounts for roughly 5–10% of diabetic cases, and individuals at increased risk can often be identified by evidence of autoimmune pathologic processes occurring at the pancreatic islets [1]. Type 2 diabetes is characterized by a progressive insulin secretory defect within a setting of insulin resistance [2]. Approximately 90–95% of diabetic cases are type 2 [1]. Management of glycemic levels in diabetic patients is critical, as persistent hyperglycemia may lend itself to a number of complications including cardiovascular disease, nephropathy, retinopathy, neuropathy, and various foot pathologies [2].

The prevalence and diagnostic criteria for diabetes are well defined. There are approximately 29.1 million people with diabetes in the United States (roughly 9.3% of the total population). Of these 29.1 million cases, around 27% or 8.1 million cases are undiagnosed [3]. Furthermore, a study funded by the World Health Organization (WHO) found that estimated 347 million people worldwide have diabetes [4]. Between 2010 and 2030, a 69% increase in the number of adults with diabetes in developing countries and a 20% increase in developed countries are predicted [5]. A diagnosis of diabetes may be confirmed through several different techniques. These diagnostic criteria include (1) hemoglobin A1c (A1c) ≥ 6.5%, (2) fasting plasma glucose ≥ 126 mg/dL (fasting is defined as no caloric intake for at least 8 hours), (3) 2-hour plasma glucose ≥ 200 mg/dL during an oral glucose tolerance test (OGTT), and (4) random plasma glucose ≥ 200 mg/dL in a patient with classic symptoms of hyperglycemia [2].

Proper glycemic control and attainment of other management goals (cholesterol, Body Mass Index (BMI), and blood pressure) are essential in prevention of long-term complications of diabetes as well as reduction of overall disease management costs [6]. In fact a recent study found that values of HbA1c that are either <6.5% or >9.0% may be associated with increased mortality within one year in clinical type 2 diabetes [7]. Tight glycemic control is becoming increasingly recognized as a perioperative goal in surgical patients [8–12]. However, there is still no overall consensus on the optimal perioperative management of the diabetic patient [13–21]. In this paper we hope to outline risk factors associated with hyperglycemia due to diabetes in the surgical patient, as well as review broad glucose management strategies during surgery as well as the pre- and postoperative stages.

## 2. Why Is Management of Diabetes Important in the Surgical Setting?

Surgical procedures may result in a number of metabolic perturbations that can alter normal glucose homeostasis. The resulting hyperglycemia due to abnormal glucose balance is a risk factor for postoperative sepsis [22], endothelial dysfunction [23], cerebral ischemia [24], and impaired wound healing [25, 26]. In addition, the stress response may also cause other diabetic pathologies including diabetic ketoacidosis [27] (DKA) or hyperglycemic hyperosmolar syndrome [28] (HHS) during surgery or postoperatively. However, recent evidence suggests that careful management of glucose levels in patients undergoing major surgeries, including cardiac [29] and orthopedic [30] procedures may minimize the aforementioned negative sequela and overall promote better outcomes. On average, diabetics require more hospitalizations, longer durations of stay, and cost more to manage than nondiabetics. The total estimated cost of managing diagnosed diabetes in 2012 was $245 billion, a 41% increase from the 2007 estimate, with the largest percentage (43% of the total medical cost) being spent on inpatient hospital care [31]. Hospitalized diabetics generally tend to be older, less active, and based on hemoglobin-AIC measurements and control their glycemic levels less aggressively [32]. Furthermore, diabetics undergo certain procedures and surgeries more commonly than nondiabetics and have increased morbidity and mortality rates when acutely compromised or ill [33–35].

Glycemic monitoring in the perioperative setting is done in a passive manner to combat any potential neuroglycopenic sequelae from underlying unrecognized hypoglycemia. Unmanaged hypoglycemia may result in a number of neurological complications including somnolence, unconsciousness, and seizures and depending on the duration, irreversible neurological insult, or death [35]. Recognition of the neurological manifestations of hypoglycemia while a patient is under general anesthesia or receiving sedatives/analgesics (with or without neuromuscular blocking agents) after completion of surgery is difficult, potentially leaving the hypoglycemic state unrecognized for a critical amount of time before proper management ensues [35]. Additionally, studies have suggested that hypoglycemia enhances morbidity/mortality in critically ill diabetic patients [36] and can prolong ICU/hospital stay [37]. In general, complications from surgical wounds are more prevalent in diabetics, and healing is impaired when glycemic levels are not well managed [38]. As diabetics tend to sustain increased perioperative morbidity and mortality, identification of diabetic patients is imperative in the surgical setting. Slightly more than a third of perioperative diabetics remain unrecognized or untreated before surgery or admittance to the ICU [39, 40]; clinicians must remain alert to properly identify diabetes, glucose intolerance, insulin resistance, and associated diabetic pathologies. Overall, with the use of careful glucose management strategies, the primary outcome measures of surgery are similar between diabetic and nondiabetic patients [41].

## 3. A Brief Summary of the Metabolic Response to Surgery and Anesthesia

The trauma associated with surgery results in increased production of stress hormones, the magnitude of which depends on the severity of the surgery or any postoperative complications. In specific, the increases in cortisol and catecholamine levels related to surgery have been well documented [42, 43]. Increased cortisol and catecholamines reduce insulin sensitivity, while heightened sympathetic activity reduces insulin secretion while simultaneously increasing growth hormone and glucagon secretion [44, 45]. In the diabetic patient, insulin production is already marginalized; the metabolic changes outlined above that occur during surgery cause a marked catabolic state. Changes in normal metabolic patterns due to surgery trigger gluconeogenesis, glycogenolysis, proteolysis, lipolysis, and ketogenesis ultimately resulting in hyperglycemia and ketosis [46].

There are a number of anesthetic drugs, each of which has a variable effect on glycemic control. Most intravenous (IV) induction agents have a relatively negligible effect on blood glucose, although a notable exception is the induction agent etomidate. Etomidate is known to cause less hypotension during induction and generally fewer hangover-like effects upon recovery [47]. Review of the etomidate mechanism shows suppressed adrenocortical function mediated by blocking the activity of 11-beta-hydroxylase, ultimately causing decreased steroidogenesis [48]. In fact, the literature reports that acute adrenocortical insufficiency and crisis may occur after a standard induction dose of etomidate [49]. However, due to diminished cortisol secretion, etomidate triggers a subsequent decrease in the hyperglycemic response to surgery [47]. Additionally, if used in high doses during surgery, benzodiazepines decrease ACTH secretion. Benzodiazepines also stimulate release of growth hormone, while reducing sympathetic stimulation [50]. Opiates given in high doses such as during the postoperative recovery phase block the sympathetic nervous system as well as the hypothalamic-pituitary axis, essentially abolishing the hyperglycemic response to surgery [51]. In vitro studies revealed that volatile anesthetic agents such as halothane and isoflurane inhibit normal insulin production triggered by glucose in a dose dependent fashion, essentially resulting in a hyperglycemic response [52, 53]. Further studies must be completed in order to understand the full clinical effects of this response in diabetic patients undergoing surgery.

Whereas most anesthetic agents cause hyperglycemia, epidural anesthesia tends to have a nominal effect on glucose metabolism [54]. Epidural anesthesia inhibits catecholamine release (irrespective of spinal segmental level), as such noradrenaline and cortisol concentrations do not increase, preventing elevation in blood glucose levels [55]. In addition, sympathetic efferent signal blockade with enhanced fibrinolytic activity blunts the surgical stress response normally responsible for hyperglycemia [56]. However, physicians must be cognizant of certain complications related to epidural and regional anesthetic use. For example, use of localized anesthesia in diabetic patients with autonomic neuropathy may result in deleterious consequences such as life-threatening hypotension. It is imperative that the anesthetic technique used allows for rapid recovery after surgery to prevent concealment of hyperglycemic or hypoglycemic coma [46].

In general, the response to neuromuscular blocking agents is normal in diabetic patients; however in patients with neuropathies or irregular transmission across the neuromuscular junction abnormalities may occur. Overall, the choice of neuromuscular blocking agent will be predicated on renal function, while anesthetic selection will be evaluated according to the degree of various systemic diseases such as diabetes, hypertension, and coronary artery disease. Finally, to insure proper postoperative management, clinicians should be aware that anesthetic agents tend to cause hyperglycemia [46].

## 4. Perioperative Assessment and Management Goals for the Diabetic Patient

Perioperative management of glucose levels revolves around several key objectives that are briefly elaborated on below:

- Reduction of overall patient morbidity and mortality [46, 57].
- Avoidance of severe hyperglycemia or hypoglycemia [46, 57].
- Maintenance of physiological electrolyte and fluid balance [46, 57].
- Prevention of ketoacidosis [46, 57].
- Establishment of certain glycemic target levels [46, 57], less than 180 mg/dL in critical patients and less than 140 mg/dL in stable patients [58].

In surgical patients, careful blood glucose control has been associated with decreased mortality [59]. Additionally, ketoacidosis in diabetic patients undergoing surgery must be avoided. Treatment of patients with DKA uses significant healthcare resources accounting for 25% of healthcare dollars spent on direct medical care for adult patients with type 1 diabetes in the United States [60]. Lastly, optimizing glucose levels according to standard hospital protocols was associated with a 25.4% reduction in perioperative complications [61]. Specific strategies for glucose management differ during surgery as well as the preoperative and postoperative stages. All of the abovementioned goals as well as distinct strategies during each phase of surgery will be addressed below. Furthermore, a graphical diagram of the perioperative timeline can be seen in Table 1.

## 5. Preoperative Glycemic Management

In patients using insulin, frequent glucose monitoring should be utilized to ensure that glucose values are within normal ranges. Patients should monitor blood glucose levels vigilantly including before and after meals as well as before sleeping. Additionally, finger stick glucose monitoring should be completed every 4 to 6 hours in any patient who is nil per os (NPO), with supplemental insulin used to correct hyperglycemia back to normal values [57]. When using supplemental-scale coverage, short-acting insulin (humulin, novolin) has a shorter duration of action than human insulin and may be given subcutaneously every 4 to 6 hours; however to prevent insulin stacking regular human insulin should not be given more than every 6 hours to correct hyperglycemia [57]. Traditionally, long-acting insulin (glargine, ultralente) is discontinued two to three days prior to surgery; glucose levels are instead stabilized by a combination of intermediate insulin (NPH) with short-acting insulin twice daily or regular insulin before meals and intermediate-acting insulin at bedtime [62]. However, if glycemic control is well managed in a patient being treated with glargine, it is acceptable to continue the same insulin regimen until the day of surgery [63]. Finally, it is important to confirm the form of diabetes present, as patients with type 1 diabetes must continue a basal rate insulin replacement preoperatively (0.2 to 0.3 U/kg/day of a long-acting insulin) [57].

Along with careful insulin regulation, there are a number of oral glycemic control drugs that should be discontinued before surgery. Biguanides (metformin) sensitize specific tissues to insulin, mediating efficient uptake of glucose in muscle and fat while preventing hepatic glucose formation. Metformin usage is discontinued before surgery in the United States and Europe due to renal function complications that may arise intraoperatively (such as hemodynamic instability or decreased renal perfusion), increasing the risk of lactic acidosis [64, 65]. Alpha glucosidase inhibitors (acarbose, miglitol) weaken the effect of oligosaccharidases and disaccharidases in the intestinal brush border, effectively lowering the absorption of glucose after meals. However, in preoperative fasting states, this drug has no effect and thus should be discontinued until the patient resumes eating [66]. Thiazolidinediones (pioglitazone, rosiglitazone) mechanism of action is similar to that of metformin and however is not associated with lactic acidosis. Nevertheless, these drugs are generally discontinued as they are not insulin secretagogues and may also cause fluid retention in the postoperative phase [57, 67]. Sulfonylureas (glibenclamide, glimepiride, and glipizide) trigger insulin production and may induce hypoglycemia in a fasting preoperative patient. If a patient has mistakenly taken a sulfonylurea on the day of surgery, the operation may still be completed; however, careful glucose monitoring is imperative and IV dextrose may be required [65, 68]. Glucagon-like peptide-1 (GLP-1) agonists (exenatide, liraglutide) are held the day of surgery as they slow gastric motility and may delay restoration of proper gastrointestinal function during recovery. Finally, because dipeptidyl peptidase-4 (DPP-4) inhibitors (sitagliptin, linagliptin) work by a glucose dependent mechanism (reducing the risk of hypoglycemia even in fasting patients) they may be continued if necessary; however, these medications primarily reduce glycemic levels after meals and their effects will be greatly marginalized in preoperative NPO patients [57].

There currently exists no evidence-based guideline dictating when to cancel surgery due to hyperglycemia. As a rule, elective surgery should not be performed on patients in a compromised metabolic state (DKA, HHS, etc.). Although no strict standard for surgical cancellation has been determined, the Yale New-Haven Hospital recommends postponing surgery if glucose is greater than 400 mg/dL. Similarly, at Boston Medical Center, it is recommended to postpone nonurgent surgical procedures if glucose is >500 mg/dL. In the event surgical cancellation is required, physicians should first manage any metabolic pathologies if present. After resolution of any underlying metabolic abnormalities, clinicians may then aim to restore blood glucose back to target range using combination insulin therapy as described above [69].

## 6. Intraoperative Glycemic Management

As described above surgical stress as well as anesthesia promotes hyperglycemia in the diabetic patient. Although there currently exists no consensus target range, in general the literature suggests keeping glucose levels between 150 and 200 mg/dL (8 to 11 mmol/L) during surgery [13–21]. Moreover, a study discovered that intraoperative hyperglycemia (glucose greater than 200 mg/dL) as well as relative normoglycemia (glucose less than 140 mg/dL) was found to be associated with significant morbidity and mortality. In fact, the study found that glucose levels ranging from 140 to 170 mg/dL had the lowest risk of adverse outcomes [70]. During surgery, glycemic levels can be sufficiently monitored by utilizing blood glucose measurement systems designed for inpatient bedside use [17, 21]. Additionally, clinicians must take the approximate length of time required to complete a procedure into consideration when determining an intraoperative glycemic control strategy. For short, minor procedures, preoperative glucose maintenance protocols may still be employed [57]. For more complex procedures, variable rate IV insulin infusion has been highlighted as a more effective method for achieving glycemic control [16, 57, 71, 72].

Regular IV insulin remains physiologically active for approximately 1 hour but has a serum half-life of 7 minutes, as such it allows for tight glycemic control that can combat unexpected changes in blood glucose effectively [57]. In patients with type 1 diabetes the insulin infusion rate begins at roughly 0.5–1 U/hour (mix 100 U short-acting insulin in 100 mL normal saline; i.e., 1 U = 1 mL), whereas infusion rates are typically increased in type 2 diabetics to approximately 2-3 U/hour or higher [20]. There are a number of both static [73, 74] and adjustable [75] algorithms that can be used to adjust the rate of insulin infusion. It should be noted that there exists a continuous Glucose-Insulin-Potassium (GIK) infusion technique, which has been supported as an inotropic and metabolic therapy in several critical disease states [76]. The proposed mechanism of GIK therapy includes lowering circulating levels and subsequent myocardial uptake of free fatty acids (which are toxic to ischemic myocardium); increased myocardial energy production through exogenous glucose; and stabilization of intracellular potassium, which may be depleted during times of myocardial ischemia [77, 78]. However, this method does not allow for individual manipulation of glucose or insulin levels if required, as such this system may be better suited for blood glucose maintenance after achievement of a specific glycemic goal [20].

## 7. Postoperative Glycemic Management

Due to postoperative complications, anesthetic side effects, or a number of other reasons, glycemic control during the postoperative stage may be difficult. The foundation of good postoperative care is based on diligent blood glucose measurement. The Society of Thoracic Surgeons as well as the AACE/ADA consensus recommended a postoperative glycemic range between 140 and 180 mg/dL [79]. However, if patients are monitored in the acute care setting after surgery due to surgical complications or various underlying comorbidities, physicians should be cognizant of the stress hyperglycemic response (averaging roughly 180–220 mg/dL) and as such develop a more tolerant glucose management strategy [80]. If blood glucose levels remain low after surgery, a dextrose infusion rate of 5–10 g of glucose per hour should prevent hypoglycemia and concomitant ketosis [13]. Additionally, if a patient is unable to tolerate oral nourishment for a prolonged period of time, total parenteral nutrition (TPN) should be considered. However, enteral nutrition should be resumed as soon as possible, due to fewer infectious complications, decreased cost, earlier restoration of normal gut function, and reduced length of hospital stay when compared to TPN [81]. Physiologic replacement of insulin can be mediated by a long-acting basal insulin dose (regardless of alimentation status), short or rapid acting insulin dose following meals, and rapid acting supplemental insulin to combat hyperglycemia if needed. Finally, according to a randomized trial conducted in 2007, in noncritically ill hospitalized type 2 diabetics, use of basal/bolus insulin protocols (as outlined below) offers significantly better glycemic control than supplemental-scale insulin alone [82].

It is important to ensure that basal insulin levels remain stable after intraoperative IV insulin is discontinued. This is especially true in type 1 diabetes, as baseline insulin values must be met to prevent diabetic ketoacidosis. The basal insulin dosage can be calculated using the “Miami 4/12” rule or approximated to 50–80% of the intraoperative IV insulin total (assuming adequate glycemic control was achieved) [57]. For patients treated with intraoperative IV insulin, it may be easiest to continue IV insulin alongside a dextrose infusion until the patient can tolerate food without difficulty. After verifying the patient is able to consume food reliably, the intravenous drips can be terminated and glycemic control procedures employed before surgery may be reinstituted [83]. Again the literature does not report a clear consensus in management, as another source instead recommends transitioning from IV to subcutaneous insulin 12 to 24 hours prior to discontinuing the drip to insure a baseline insulin concentration in type 1 diabetics (significantly reducing the chances of diabetic ketoacidosis) and allowing for heightened glycemic control in type 2 diabetic patients [57].

Postprandial related insulin requirements must be tailored to the mode in which the patient is receiving nutrition. Moreover, patients should be given instructions on how to initiate subcutaneous insulin supplementation in the event of hyperglycemia. For clinicians, supplemental insulin compensation for hyperglycemic patients can be approximated by dividing the total daily insulin (TDI) dose by 30 for every 50 mg/dL (3 mmol/L) above the glycemic goal [83]. Take a patient with a TDI dose of 150 U with a blood glucose reading of 350 mg/dL. Subtracting the upper end of a normal glucose measurement (200 mg/dL) from the patients reading and diving by 50 mg/dL yields 3 [(350 − 200)/50 = 3]. Simply multiply this number by the TDI/30 (150/30 = 5) to determine the patient requires an additional 15 U of rapid acting insulin to restore blood glucose levels back into target range. Finally, glucose measurement during the perioperative period can generally be completed by either central-laboratory-device (CLD) or point-of-care (POC) devices [84]. Multiple studies recommend avoiding the POC device for glucose management during the perioperative period, instead favoring the use of CLD blood glucose measurements [84, 85]. A summary of key checkpoints regarding pre-, intra-, and postoperative glycemic control can be reviewed in Table 1.

## 8. Special Operative Conditions

There are a number of special operative conditions that should be taken into account when determining a glucose management plan. For minor outpatient surgeries, type 1 or type 2 diabetes can be managed by IV infusion or subcutaneous insulin strategies. Furthermore, type 2 diabetic patients who are taking oral glycemic control agents should follow similar management guidelines as described above [20]. For emergency surgery situations, blood glucose should be monitored frequently. Physicians should also note when the last dose of a sulphonylurea drug was taken, as progressive absorption may disturb glycemic control [46, 86]. Insulin requirements are generally much higher in cardiac procedures; recent studies suggest improved patient outcomes with tight glycemic control during and after cardiac surgery [87]. Finally, perioperative blood glucose levels must be carefully monitored in patients undergoing cesarean section. Hyperglycemia should be avoided during cesarean section to reduce the risk of neonatal hypoglycemia or wound infections in the mother. Before induction of labor, patients should follow their normal diabetic regimen; however if labor is prolonged and blood glucose levels fall below 100 mg/dL, a 5% dextrose infusion should be initiated [88].

## 9. Conclusion

A number of protocols defining perioperative glycemic control have been described in the literature. While clinical judgment must still be used to assess specific changes, we hope this paper has provided greater insight into the overall goals of glucose management during pre-, intra-, and postoperative periods. Healthcare providers should remember that glucose homeostasis during the perioperative period is extremely variable; blood glucose levels as well as electrolyte and acid-base status should be carefully monitored. Physicians should be mindful of a patient's normal diabetic regimen, and after making all necessary changes during the perioperative period, aid the patient's transition back to their normal glycemic management protocol. In closing, through careful perioperative glucose management, surgical complications as well as hyper- or hypoglycemic sequelae can be reduced, ultimately improving patient morbidity and mortality.

## Figures and Tables

**Table 1:** Broad management goals across the perioperative timeline. Overall goals: (i) reduce patient morbidity and mortality, (ii) avoid clinically significant hyper- or hypoglycemia, (iii) maintain acid/base, electrolyte, and fluid balance, (iv) prevent ketoacidosis, and (v) establish blood glucose measurements less than 180 mg/dL in critical patients and less than 140 mg/dL in stable patients.

Preoperative management key points	Intraoperative management key points	Postoperative management key points
(i) Verify target blood glucose concentration with frequent glucose monitoring (ii) Use insulin therapy to maintain glycemic goals (iii) Discontinue biguanides, alpha glucosidase inhibitors, thiazolidinediones, sulfonylureas, and GLP-1 agonists (iv) Consider cancelling nonemergency procedures if patient presents with metabolic abnormalities (DKA, HHS, etc.) or glucose reading above 400–500 mg/dL	(i) Aim to maintain intraoperative glucose levels between 140 and 170 mg/dL (ii) Physicians must take length of surgery into account when determining an intraoperative glucose management strategy (iii) For minor surgery, preoperative glucose protocols may be continued (iv) IV insulin infusion is being promoted as a more efficient method of glycemic control for longer or more complex surgeries	(i) Target postoperative glycemic range between 140 and 180 mg/dL (ii) In the event a patient is hypoglycemic after surgery, begin a dextrose infusion at approximately 5–10 g/hour (iii) Ensure basal insulin levels are met, especially in type 1 diabetic patients (iv) Postprandial insulin requirements should be tailored according to the mode in which the patient is receiving nutrition (v) Supplemental insulin can be used to combat hyperglycemia and restore blood glucose values back to target range

Please note that the information presented in this table has been referenced in the text.
